# Splendid but Hopeless

**Published:** 1989-08

**Authors:** J. L. Burton

**Affiliations:** Consultant Dermatologist, Bristol Royal Infirmary


### Splendid but Hopeless

**J. L. Burton**
Consultant Dermatologist, Bristol Royal Infirmary

As the invited Guest Lecturer, I was to be accommodated in the Indian Government House on the outskirts of the city. At first the battered taxi could only crawl through the crush of pedestrians, beggars, handcarts, bicycles, cows, holy men, pilgrims and mobile wood-stacks that clogged the streets of the city centre, Gradually however the crowd thinned slightly, the driver put his foot down and after a certain amount of stuttering and coughing, the ailing vehicle slowly built up speed. Thereafter the driver's technique for dealing with any obstacle was to blow his horn and accelerate towards it. Miraculously the obstacle would melt away at the very last moment, or if it didn't, we simply swerved around it without slackening speed. Considering the alarming degree of play in the steering wheel, this was quite a feat. I began to believe that the plastic Hindu gods which dangled in profusion from the windscreen, partially obscuring the driver's view, really were all-powerful, or else the hazards (old ladies, toddling babies, dogs and all) were mirages.

At length however we succeeded in knocking a man off his bicycle. My driver leapt out, and paused only long enough to ascertain that the man was not seriously hurt before we sped off again, leaving the cyclist shaking his head over his badly buckled wheel. Shortly afterwards the road ahead was blocked by a large red and white mound, half-hidden under a flapping mass of vultures. The lorry which had caused this carnage lay wrecked in a ditch nearby. The accident had apparently happened a day or two previously, but neither the lorry nor the bullock carcase could be moved until compensation had been paid to the bullock's owner.

We travelled several miles through the inner suburbs, a depressing area of endless repair shops of various kinds, half-built houses, bill-boards and powerlines. Eventually the bill-boards disappeared, the houses began to be more widely spaced, the roads became even more rutted, and we entered the residential area favoured by the affluent businessmen, lawyers, doctors, dentists and colonels. Each large villa with its well-kept garden was distinguished by a smart brass name-plate on the white-painted gatepost. Most gateposts appeared to have a dirty bundle of rags piled against them, but these turned out to be the gate-keepers, who drowsily squatted beside the gates all day, waiting to fling them open and kow-tow at the approach of the villa's illustrious owner.

At one such gate we swept up the drive, horn blaring, and two gardeners ran through the bushes to stand by the side of the drive and salute me with the traditional 'namaste' greeting of bowed head and hands held upright, palms together. Between the bushes I glimpsed a peacock on the lawn and feeling like Lord Mountbatten, I alighted before a magnificent stone bungalow, the colonnades around its huge bay-windows half hidden beneath festoons of blossom-laden creepers. And on the steps, ranged in order of seniority, were three of the scruffiest ruffians I have ever clapped eyes on.

The huge bedroom was furnished in an ornate Edwardian style, with heavy curtains draped around the bed in addition to the usual mosquito nets. There were no windows and the room was dimly lit by a single naked light bulb. The light switch fitted uncomfortably into an irregular cavity in the plaster-work with its bare wires visible in the recess, and the whole thing wobbled dangerously whenever the light was switched on.

A door at the far end of the bedroom led into an even more cavernous bathroom, one corner of which was occupied by a metal bath which could have accommodated an entire football team. The floor was concrete, the small mirror was cracked, the toilet cistern was rusty, the sink lacked a plug and indeed the whole ambience was strongly reminiscent of the changing-room facilities offered by the Pinxton Rovers Second team of my youth. There was of course no toilet paper, and no hot water, but I discovered later that hot water could be brought in by prior arrangement with the Three Ruffians, who made repeated trips to carry it in by the bucket-full, coolie style, until Sahib deemed the water level to be satisfacotry. As I re-entered the bedroom and closed the door, something green plopped onto the floor and scuttled under the drapes of the bedspread.

The dining-room was wood panelled, but the atmosphere was curiously spartan, like a run-down Youth Hostel. The only picture, stuck on a piece of cardboard, had apparently been clipped from a woman's magazine and framed with passe-partout. As I entered the room, the Senior Ruffian appeared immediately, obsequiously pulled out a chair for me and dusted a place for me at the table, using a grey rag of uncertain provenance, which he flapped in the Indian manner, so that the crumbs and debris were rearranged rather than removed.

"Vot you vant for breakfast sah?" he asked politely. After some discussion, which was prolonged by our mutual difficulties with pronunciation and comprehension, I gathered that I

could have absolutely anything I liked so long as it was what they had. On this particular day, what they had (as on every subsequent day) was cornflakes, bread, butter, jam, tea, tomato ketchup and salt but "Ve are regretting sah, no sugar". The cornflakes were of an interesting vintage. At first I thought they had curious little black raisins in them, but they turned out to be desiccated flies.

The Three Ruffians arranged themselves strategically around the table and watched me intently with dark spaniel eyes, eager to push the cornflakes, the jam or the butter within my reach at my slightest whim, or even if absolutely necessary, to fetch a knife and spoon.

My request for milk to go with the cornflakes clearly came as a surprise, but the system coped well. The Senior Ruffian translated my request into Hindi and relayed it to the Middle Ruffian, who passed it onto the Junior Ruffian who dashed out into the courtyard. I heard the shouted order volley down the chain of command into the middle distance until it reached the cow, buffalo or whatever. Ten minutes later a jug of warm frothy milk was laid before me and I thanked the Lord for the B.C.G. Clearly such a coup was not accomplished every day "You are vanting anything else sah?", was accompanied by the broadest of smiles and that curious head-shaking movement which implied that absolutely nothing was impossible for this team. I considered a kipper, but thought of the distance to the sea.

After breakfast I strolled in the garden. Flowers abounded, of every hue, and the warm air was heavy with their scent. Chipmunks scampered up the trees ahead, and as I walked towards them I disturbed two large brown birds, which erupted into the clearing with a wild beating of wings, and for a second or two the whole sky seemed filled with electric-blue lightning.

From beyond the garden came the sound of children's laughter, and the splashing of water. Willowy girls in vivid saris filed away from the water tank, their bangles and the brass water-pots sparkling in the sunshine. In the distance woodsmoke smudged the sky and a donkey brayed, and in the foreground a dead dog decayed.

